# Winter Cultivation and Nano Fertilizers Improve Yield Components and Antioxidant Traits of Dragon’s Head (*Lallemantia iberica* (M.B.) Fischer & Meyer)

**DOI:** 10.3390/plants9020252

**Published:** 2020-02-16

**Authors:** Vida Mohammad Ghasemi, Sina Siavash Moghaddam, Amir Rahimi, Latifeh Pourakbar, Jelena Popović-Djordjević

**Affiliations:** 1 Department of Plant Production and Genetics, Faculty of Agriculture, Urmia University, Urmia 5756151818, Iran; vidamohamadghasemi.1397@gmail.com (V.M.G.); e.rahimi@urmia.ac.ir (A.R.); 2 Department of Biology, Faculty of Science, Urmia University, Urmia 5756151818, Iran; l.pourakbar@urmia.ac.ir; 3 Department of Food Technology and Biochemistry, Faculty of, University of Belgrade, 11080 Belgrade, Serbia; jelenadj@agrif.bg.ac.rs

**Keywords:** *Lallemantia iberica*, nanofertilizer, chelated nano iron, essential oil, TPC, TFC

## Abstract

Balangu (*Lallemantia* sp.) is a medicinal herb with a variety of applications, all parts of which have economic uses, including leaf for extraction of essential oils, as a vegetable and potherb, seed for extraction of mucilage and edible or industrial oil. To investigate the effect of cultivation season and standard chemical and nano fertilizers (n) on the yield components and antioxidant properties of Dragon’s head, a factorial experiment based on randomized complete block design was conducted with 12 treatments and three replications. Experimental treatments consisted of two seasons (spring and winter cultivation) and six levels of fertilizer (control, NPK-s, NPK-n, Fe-chelated-n, NPK-n + Fe-chelated-n, NPK-s + NPK-n + Fe-chelated-n). The traits included grain yield per plant, essential oil percentage and yield, mucilage percentage and yield, antioxidant properties in the seeds and leaves, including total phenols and flavonoids content, DPPH radical scavenging, and nitric oxide and superoxide radical scavenging. The results showed that winter cultivation had a noticeable advantage over spring cultivation across all of the traits. The highest grain yield per plant was obtained in winter cultivation using NPK-n + Fe-chelated-n fertilizer treatment. The highest essential oil percentage was in NPK-n + Fe-chelated-n. The highest mucilage percentage was observed in NPK-s + NPK-n + Fe-chelated-n fertilizer treatment, which was not statistically different to NPK-n + Fe-chelated-n treatment. The combined effects of winter cultivation and NPK-n + Fe-chelated-n fertilizers resulted in improving antioxidant activity traits. Overall, the results indicated that the combination of winter cultivation and NPK-n + Fe-chelated-n fertilizers are the most appropriate treatment to acquire highest qualitative and quantitative yield of Dragon’s head, in the Azerbaijan region (Iran).

## 1. Introduction

The use of medicinal plants and plant-derived medicine is increasing in different countries at a growing pace, mainly because the effectiveness of these substances has been scientifically proven and they are popular in most human societies. Due to growing concerns over the side-effects of chemical medications and the futility of some of them in their long-term use, attention has been drawn to the use of natural compounds as an alternative or supplement. The use of plants by humans as medicine can be traced back to very early civilizations [1]. The willingness to produce medicinal and aromatic herbs and the demand for natural products, especially those grown in ecological cultivation conditions, are increasing in the world [2].

The family Lamiaceae is one of the biggest and most famous plant families, with high biodiversity around the world. The medicinal and aromatic members of this family are regarded as a key genetic reserve of plants due to their high ecological adaptability with diverse climates. Also, because of their aromatic compounds, they are widely used in the cosmetic, health, medicinal, and food industries [3]. *Lallemantia iberica* as an annual–biennial herb is recognized commonly as dragon’s head and is a species of flowering plant in the mint family, Lamiaceae. Its extracts have pharmaceutical and medicinal properties. It includes terpenoids, flavonoids compounds, and essential oil [4]. The leaves, oil, and seeds of *L. iberica* can be consumed, and when the plant is grown as a vegetable for fresh consumption, it should be harvested before flowering [5]. The physiological and morphological traits of this plant species are affected by the sowing season. Sowing season mostly affects the length of the vegetative and reproduction phase, crop harvest, yield, and quality. Sowing season and time due to its relationship with day length, temperature, and relative humidity have a great impact on the quality and yield components of plants. Sowing season is one of the most important management factors (controlling the damage of pests, diseases, weeds, and cold temperatures, etc.) for producing a high value product [6]. The selection of sowing season is influential in controlling the damage of pests, diseases, weeds, and cold temperatures, and matches the plant’s growth period, especially in sensitive development phases like flowering, with suitable temperatures [7].

Given the excessive consumption of chemical fertilizers and the resulting contamination of groundwater and soil salinization, the use of nano-fertilizers is highly efficient. Rawat et al. revealed that nano-sized fertilizers have the potential to be employed as a plant growth promoter and can enhance plant gas exchange and root efficiency [8]. Moreover, nano fertilizers, due to the slow and controlled release of nutrients, are capable of increasing the availability of nutrients in the rhizosphere zone [9]. Research has shown that soils have a deficiency in macroelements, especially nitrogen (N), phosphorus (P), and potassium (K), and these are more commonly found as components of commercial fertilizers, compared to the other essential elements [10]. Nano-Fe fertilizer has several advantages over other Fe fertilizers, such as increasing plant metabolism, and improving nutrient uptake efficiency and mobilization [11]. Basically, in the arid and semi-arid climates of Iran, the majority of farmers apply different types of Fe chelates, however, their use is still in question. Nanoparticles are assumed to be one of the best choices for these areas. The effect of nano Fe chelated on growth and development of medicinal plants and herbs has been widely studied. But all aspects of plant responses to iron nanoparticles are still unclear [12].

The present study aimed to shed light on the effects of the sowing season (winter and spring) and the integrated use of chemical fertilizer and nano-fertilizers on the quantitative traits, essential oil content, mucilage, and antioxidant properties of *L. iberica.*

## 2. Results

### 2.1. Seed Yield per Plant

**Figure 1:**
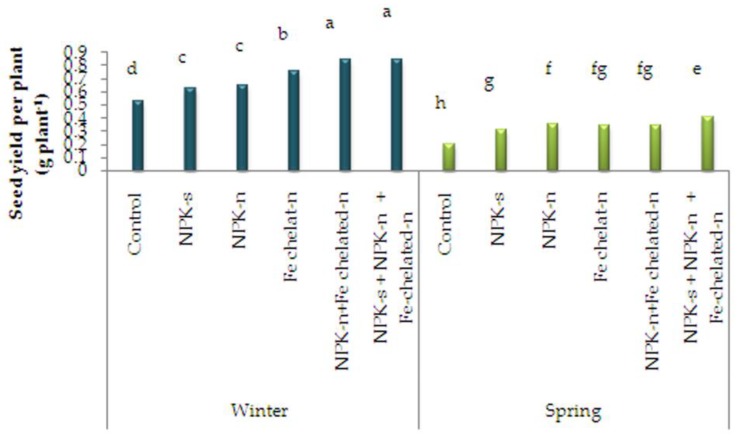
The interactive effect of sowing season and fertilizer application on seed yield of *L. iberica.* Different letters mean significant differences according to Duncan’s multiple range test at *p* < 0.05.

The comparison of data means showed that the plants treated with NPK-n + Fe-chelated-n and sown in winter showed the highest seed yield per plant (0.84 g plant^−1^) and the control plants sown in spring produced the lowest one (0.21 g plant^−1^, Figure 1).

### 2.2. Mucilage Percentage and Yield

**Figure 2:**
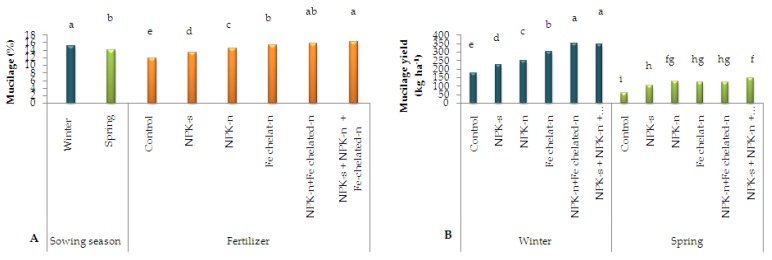
The simple effect of sowing season and fertilizer application on mucilage percentage (**A**) and mucilage yield (**B**) of *L. iberica* Different letters mean significant differences according to Duncan’s multiple range test at *p* < 0.05.

Based on the results of analysis of variance (ANOVA), the simple effects of sowing season and nano fertilizers had significant (*p* < 0.01) effects on mucilage percentage and the interactive effects were not significant. The comparison of means indicated that the highest mucilage percentage was related to the incorporated NPK-s + NPK-n + Fe-chelated-n fertilizer (16.66%) and winter sowing (15.05%), and the lowest was obtained from the control (11.14%) and spring sowing (13.9%, Figure 2A). ANOVA revealed significant differences in mucilage yield, as influenced by the interactive effect of sowing season and nano fertilizers. According to the comparison of means, the highest mucilage yield was 348.49 kg, obtained from the plants sown in winter and fertilized with NPK-n + Fe-chelated-n, and the lowest was 61.00 kg, obtained from the control spring-sown plants (Figure 2B).

### 2.3. Essential Oil Percentage and Yield

**Figure 3:**
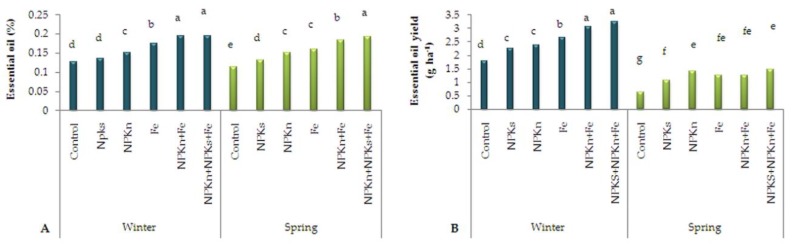
The interactive effect of sowing date and fertilizer application on essential oil percentage (**A**) and essential oil yield (**B**) of *L. iberica.* Different letters mean significant differences according to Duncan’s multiple range test at *p* < 0.05.

The results of ANOVA showed that the interactive effect of sowing season and nano fertilizers was significant on the essential oil percentage of *L. iberica*. According to the comparison of means, the highest essential oil percentage (0.194) was obtained from the interaction of soil-incorporated NPK-s + n-NPK-n + Fe-chelated-n and winter sowing and the lowest (0.11) from the interaction of control and spring sowing (Figure 3A). Essential oil yield was also significantly influenced by the interaction of sowing season and fertilizer, so that the highest essential oil yield of 3.24 mg/ha was related to the interaction of soil-incorporated NPK-s + NPK-n + Fe-chelated-n fertilizer and winter sowing, and the lowest (0.62 mg ha^−1^) was related to spring sowing (Figure 3B).

### 2.4. Antioxidants in Seed and Leaf Extracts

**Figure 4:**
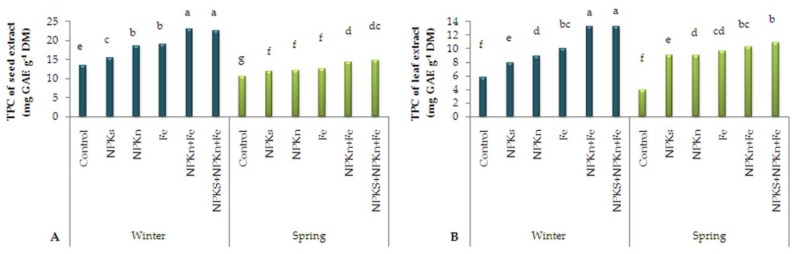
The interactive effect of sowing date and fertilizer application on total phenol content of seed extract (**A**) and leaf extract (**B**) of *L. iberica.* Different letters mean significant differences according to Duncan’s multiple range test at *p* < 0.05.

The statistical analysis of total phenols content (TPC) of the seed extract of *L. iberica* showed the highest phenol content was 23.02 mg gallic acid (GAE) per g dry matter (DM) related to the interaction of NPK-n + Fe-chelated-n and winter sowing and the lowest was 10.43 mg GAE g^−1^ DM related to the interaction of the control and spring sowing (Figure 4A). With respect to the TPC of leaf extract, the highest (13.26 mg GAE g^−1^ DM) was measured in the winter-sown plants treated within corporated NPK-s + NPK-n + Fe-chelated-n and the lowest (3.85 mg GAE g^−1^ DM) in the spring-sown plants that were not treated with any fertilizers (Figure 4B).

**Figure 5:**
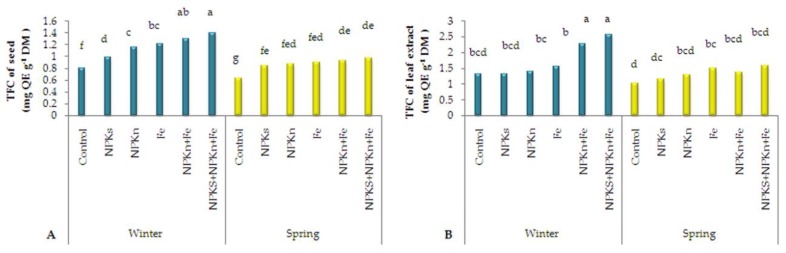
The interactive effect of sowing season and fertilizer application on total flavonoid content of seed extract (**A**) and leaf extract (**B**) of *L. iberica.* Different letters mean significant differences according to Duncan’s multiple range test at *p* < 0.05.

The statistical examination of total flavonoid content (TFC) of the seed extract indicated that the interaction of incorporated NPK-s + NPK-n + Fe-chelated-n and winter sowing was related to the highest total flavonoid content of 1.40 mg quercetin equivalent (QE) per g DM and the interaction of the control and spring sowing was related to the lowest one of 0.64 mg QE g^−1^ DM (Figure 5A). With respect to the leaf extract, it was shown than the interaction of NPK-s + NPK-n + Fe-chelated-n and winter sowing had the highest flavonoid content, 2.55 mg g^−1^, and the spring-sown control plants had the lowest, 1.03 mg g^−1^ (Figure 5B).

**Figure 6:**
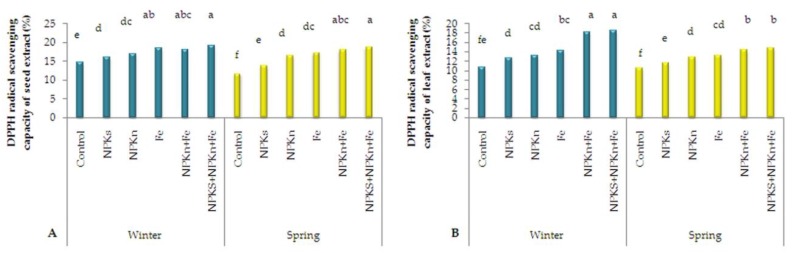
The interactive effect of sowing season and fertilizer application on DPPH free radical scavenging capacity of seed extract (**A**) and leaf extract (**B**) of *L. iberica.* Different letters mean significant differences according to Duncan’s multiple range test at *p* < 0.05.

The total flavonoid content results for the seed extract revealed that the interaction of incorporated NPK-s + NPK-n and the spring-sown control plants had the lowest DPPH (1,1-diphenyl-2-picrylhydrazyl) radical scavenging capacity of 11.74% (Figure 6A). The highest DPPH radical scavenging capacity in leaf extracts (18.48%) was observed in the winter-sown plants treated with incorporated NPK-s + NPK-n + Fe-chelated-n, and the lowest (10.5%) was for the control spring-sown plants (Figure 6B).

### 2.5. Nitric Oxide Radical Suppression Capacity of Seed and Leaf Extracts

**Figure 7:**
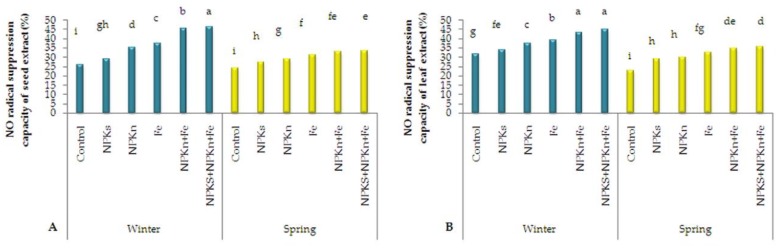
The interactive effect of sowing season and fertilizer application on nitric oxide suppression capacity of seed extract (**A**) and leaf extract (**B**) of *L. iberica.* Different letters mean significant differences according to Duncan’s multiple range test at *p* < 0.05.

The nitric oxide (NO) radical suppression results for the seed extract of *L. iberica* (Figure 7A) showed that the highest NO suppression percentage (46.34%) was obtained from the winter-sown plants fertilized with incorporated NPK-s + NPK-n + Fe-chelated-n and the lowest (24.12%) from the control spring-sown plants. At lower rates, the seed extract of *L. iberica* had a weaker nitric oxide radical scavenging rate, which increased with the increase in extract concentration. The statistical analysis of NO scavenging percentage in the leaf extract revealed that the winter-sown plants treated with incorporated NPK-s + NPK-n + Fe-chelated–n had the highest (44.88%) and the control spring-sown plants had the lowest (22.9%) (Figure 7B).

### 2.6. Superoxide Radical Suppression Capacity of Seed and Leaf Extracts

**Figure 8:**
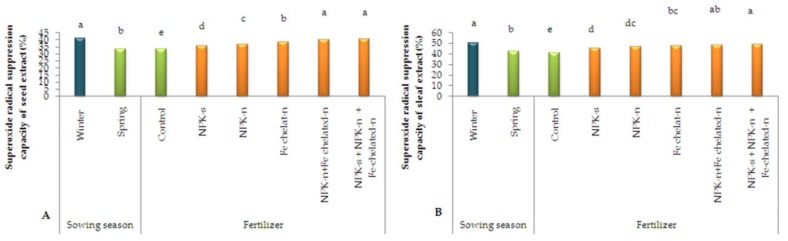
The simple effect of sowing season and fertilizer application on superoxide radical suppression of seed (**A**) and leaf extract (**B**) of *L. iberica.* Different letters mean significant differences according to Duncan’s multiple range test at *p* < 0.05.

Based on the results of the statistical examination of the superoxide radical suppression capacity of seed extract (Figure 8A), the two factors of sowing season and fertilizer choice separately influenced this trait significantly. The highest superoxide radical suppression capacity of 40.41% was related to the treatment of incorporated NPK-s + NPK-n + Fe-chelated-n and the lowest of 33.44% to the control. It was also 41.3% for winter sowing and 33.6% for spring sowing. Similarly, the examination of this trait in leaf extract indicated that incorporated NPK-s + NPK-n + Fe-chelated-n and control had the highest and lowest capacities of 49.08% and 40.88%, respectively. Also, winter sowing and spring sowing exhibited capacities of 50.55% and 42.09%, respectively (Figure 8B).

## 3. Discussion

### 3.1. Seed Yield per Plant

Previous research has established that when plants are sown early (in winter), the variations in day length and relative diurnal temperature regime provides better conditions for the growth and establishment of more vigorous plants. As such, their seed yield is enhanced. Also, their spring growth starts earlier and the period from sowing to flowering is shortened, avoiding the coincidence of a seed filling period with high environmental temperatures, lower plant height, and the loss of seed yield [13,14]. The interaction of N and macro-P and nano K fertilizers applied at the rate of 100 kg ha^−1^ and sprayed at the rate of 2:1000 improved the seed yield of *L. iberica*; similarly, the application of Fe-chelated-n fertilizer improved leaf, shoot and pod weight, thereby enhancing seed yield of soybeans [15]. Our results revealed that incorporated NPK-s + NPK-n + Fe-chelated-n had an impact similar to NPK-n + Fe-chelated-n. So, our results suggest it is enough to apply NPK-n + Fe-chelated-n, and soil-incorporated NPK can be eliminated. The spring-sown plants failed to produce as high a yield as the control treatment of the winter sowing, even when they were treated with fertilizers. So, it is recommended to sow *L. iberica* in winter.

### 3.2. Mucilage Percentage and Yield

Previous studies have indicated that *Psyllium* and *L. iberica* sown in February have a longer growth period than those sown in March, so they are in a better place to synthesize seed components, especially mucilage [14]. As such, it was found that delayed sowing reduced seed yield and mucilage yield since the growth period was shortened and the flowering and seed filling periods coincided with hot summer temperatures [15]. The mucilage percentage of *L. iberica* was increased in winter sowing treated with NPK fertilizer and the simultaneous application of P and N influenced mucilage yield [15,16,17]. In a study on *Psyllium*, Ramrudi and co-workers reported that the foliar application of micronutrients enhanced mucilage yield significant compared to the control [18]. Likewise, some researchers have attributed the higher seed and mucilage yield of psyllium to the application of fertilizers. Thus, it was shown that the higher mucilage yield was associated with the higher seed yield and mucilage percentage under the influence of optimal sowing season and fertilizer treatment. It is therefore recommended to use winter sowing and apply NPK-n + Fe-chelated-n in order to achieve the highest mucilage percentage and yield at lower costs.

### 3.3. Essential Oil Percentage and Yield

Thus far a number of studies have demonstrated that the chemical composition of essential oils varies with geographical location, growing region, soil type, climate, altitude from sea level, and water availability. Even season, e.g., before or after flowering and the hour at which setting is done, affects the chemical composition of essential oils [19]. Our results are consistent with Salamon and co-workers who reported that the quantity and quality of *L. iberica* essential oils were influenced by genotype, but climatic conditions and the interactive effect of plant and environmental conditions also influenced this trait [20]. Furthermore, plants had more of a chance for organic matter accumulation in the first sowing date compared to the second. The results obtained for the effect of sowing date on *L. iberica* and the production of more essential oil are in agreement with that previously reported about chamomile, *Dracocephalum moldavica* L., and fennel [21,22,23]. Winter sowing improves essential oil yield by increasing flower yield per unit area. The interaction of sowing season and N-containing nano fertilizer improved essential oil yield of safflower. Likewise, the application of micronutrients like Fe and the integrated use of nano P and K fertilizer and vermicompost increased essential oil percentage and yield of mint and savory [24,25,26]. We found that incorporated NPK-s + NPK-n + Fe-chelated-n had an effect similar to NPK-n + Fe-chelated-n in increasing the number of essential oil-producing glands. Hence, it is recommended to eliminate incorporated NPK-s and consume only NPK-n + Fe-chelated-n with winter sowing.

### 3.4. Antioxidants in Seed and Leaf Extracts

Several studies have revealed that these fertilizer treatments improve this trait compared to the control, and the integrated treatments were more effective than the simple treatments, which can be attributed to the positive effect of these fertilizers on the physical and chemical features of the soil. The addition of nano Fe-chelate to integrated fertilization regimes increased phenol synthesis in *L. iberica* plants by inhibiting the conversion of hydrogen peroxide to free radicals [26,27]. Research also shows that there is a direct relationship between the content of phenol compounds and antioxidant activity [28,29,30]. The higher content of phenol compounds as a free radical scavenger is the main reason for the higher antioxidant activity of the plant extracts. It has been documented that growth and nutritional conditions bring about various phytochemical changes in plants. For example, the application of K and N fertilizer increased phenol compounds in basil and tea leaves or the foliar application of Fe, Zn and Cu enhanced borage herb total phenols. Fertilizers can be applied as a soil enhancer, so that nutrients are absorbed by the plant roots, or applied as a foliar application, in which they are absorbed through the leaves, or in a combined method. Research has shown that foliar application had more beneficial effects than soil application. Because nanoparticles have high surface/volume ratios, they subsequently cause higher leaf absorption properties (than soil application) that increase biochemical activities and reactivity [31,32,33,34].

Flavonoids are a large group of phenol compounds in plants that inhibit lipid oxidation by scavenging free radicals and/or mechanisms for extinguishing singlet oxygen [35]. It was reported in the literature that the sowing dates of October and March increased quantitative and qualitative yields of intercropped peppermint and fenugreek (essential oil, phenol, flavonoid, and antioxidant capacity) as compared to plants sown in May, because flavonoids are secondary metabolites that are influenced by environmental conditions including sowing date [36], a fact that was also observed in our study. The effect of nano P fertilizer, salicylic acid, and foliar application of macro NPK fertilizers was also found to be significant on flavonoid compounds of garden sage and two olive cultivars [37,38]. Total flavonoid content and antioxidant activity were higher in leaf extract than in stem extract. Also, total flavonoid content was the highest during flowering and then during seed-setting periods. These differences were observed between leaves and seeds in our experiment, reflecting significant differences in the quantity and quality of antioxidant and phytochemical properties in different plant parts [39]. So, it is suggested to stop using soil-incorporated NPK in both sowing seasons to reduce the costs and move towards sustainable agriculture.

Nano complex of Fe could increase antioxidant activity in *Portulaca oleracea.* These nutrients play a key role in cellular mechanisms, osmotic regulation, and enzyme synthesis, and provide a large number of biochemical constitutions for many metabolic pathways, to improve plant quality and quantity [40,41]. There is a direct relationship between phenol compounds and DPPH radical scavenging activity, so that when the phenol compound content is increased, DPPH radicals are scavenged at a faster pace. The purple-colored DPPH acts as an oxidizer in oxidation and reduction reactions. DPPH free radicals are mainly composed of stable nitrogen and can be used to specify the scavenging of free radicals through antioxidant activities [42]. Important research findings have revealed that winter sowing of *Echinacea angustifolia* increased root volume compared to its spring-sown counterparts, and the measurement of antioxidant activity with DPPH showed the increased antioxidant capacity of the plants [42]. Similarly, we observed that the sowing season was effective in *L. iberica*’s capacity to improve DPPH radical suppression and increase antioxidant activity. Antioxidant capacity of the fruit extract of apricot and *Cassia fistula* was measured at different rates of Au, Ag and ZnO nano fertilizers. Based on the results of the color change mechanism, free radical reduction, and electron transfer, DPPH radical suppression was higher at higher fertilizer levels [43,44,45]. Consequently, antioxidant capacity was improved. Likewise, the application of fertilizers improved DPPH radical suppression capacity of *L. iberica* in our study. This capacity was better in leaf extract of the winter-sown plants treated with NPK-n + Fe-chelated-n, so it is recommended to use a combination of these two treatments to gain quality of yield. Nanoparticles stimulate plant antioxidant defense by oxidative stress through interaction with the cell membrane, as well as biomolecules [46]. Chandra et al. described that tea leaves treated with nanoparticles displayed higher activity of enzymatic and non-enzymatic antioxidant defense parameters such as peroxidase, polyphenol oxidase, superoxide dismutase, catalase, and phenol content [47].

### 3.5. Nitric Oxide Radical Suppression Capacity of Seed and Leaf Extracts

Treatments that showed higher suppression capacity in winter sowing performed moderately in spring sowing, revealing the impact of the sowing season. Nitric oxide radical suppression is directly related to phenol compounds of the plant. These compounds are a function of process, genotype, harvest date, growth conditions, and nutrition. Thus, the change in phenolic concentration changes nitric oxide radical suppression percentage. As such, research on the effect of harvest time on the antioxidant properties of licorice showed that the roots of the plants harvested in October had higher free radical suppression capacity, reducing capacity, and anti-oxidant capacity than those harvested in January [48]. This is evident in our research too. A study on two cultivars of marigold cultivars treated with nano Fe-chelate indicated that the fertilizer improved non-enzymatic antioxidant properties [49,50]. Phenolic compounds are known as an admirable antioxidant because of their hydrogen donating behavior, which neutralize oxidative stress. They have the ability to interfere with the process of free radical generation and act as metal chelators by stimulating antioxidant enzymes [51,52,53]. Pourakbar and Adlifard assessed total antioxidant activity, total phenol content, and total flavonoid content of different parts of grape (leaf, unripe grape, raisin, and syrup) and reported that leaves had higher total phenol and flavonoid content than unripe fruits, ripe fruits, dry fruits, and syrup, so they had the highest DPPH, superoxide, and nitric acid scavenging capacity and the highest capacity to suppress lipid peroxidation [54]. Between the two seasons studied, winter sowing performed better and the effect of soil-incorporated NPK-n + NPK-n + Fe-chelated-n was remarkable, but did not differ significantly from NPK-n + Fe-chelated-n. Nano particles are tiny in size, which gives them unique physical and chemical properties with ultra-high absorption. They are able to hold abundant ions because of their high surface area, and slowly release them in appropriate time in accordance with crop demand that results in plant photosynthesis and growth enhancement [55,56]. Thus, to accomplish the goals of sustainable agriculture and to save in costs, it is recommended to apply NPK-n + Fe-chelated-n and spring sowing.

### 3.6. Superoxide Radical Suppression Capacity of Seed and Leaf Extracts

According to Motlagh et al. [57] the sowing date had a significant effect on the antioxidant properties of roselle by influencing the length of different vegetative and reproductive growth phases and growing degree days [57]. As a result of the phenol content of the seed extract, hydrogen peroxide radical scavenging activity was increased. The synthesis of secondary metabolites and antioxidant capacity can be enhanced by substrate nutrient management and manipulation of environmental conditions [58,59]. Literature data have shown that the total antioxidant activity of savory varies with fertilizer levels [60,61]. Trace elements like Fe, Zn, Cu, Mg, and Mn play a significant role as cofactors in the structure of a group of antioxidants, so their deficiency reduces the activities of antioxidants [62]. The lack of any significant differences between leaves and seeds of borage in superoxide radical scavenging capacity was reported in the literature [62,63]. However, the seed extract exhibited higher hydrogen peroxide radical scavenging activity due to its higher phenol content. The results proved the superiority of winter sowing and incorporated NPK-s + NPK-n + Fe-chelated-n, but this fertilization regime did not differ from NPK-n + Fe-chelated-n significantly, so it is more cost-effective to apply the latter regime and eliminate soil-incorporated NPK-s.

## 4. Materials and Methods

This study was conducted in the research farm of Faculty of Agriculture, Urmia University, located in Western Azerbaijan province, Iran (Long. 45°10′ E., Lat. 37°44′ N., Alt. 1338 m.) in the crop season of 2017–2018. Table 1 and Table 2 show the soil characteristics and climatic conditions of the research farm, respectively. Fertilizer requirements were calculated based on the results of soil analysis and fertilizer recommendations and they were incorporated with soil before sowing.

The study was carried out as a factorial experiment based on a randomized complete block design with three replications. The factors included sowing season at two levels (winter and spring) and fertilizer source at six levels (control, incorporated NPK-s, NPK-n, Fe-chelated-n, NPK-n + Fe-chelated-n, and incorporated NPK-s + NPK-n + Fe-chelated-n, where n-nano fertilizer, s-standard fertilizer. The characteristics of Nano Fertilizers provided by the Khazra company were as follows: 9% Chelated Nano Iron: contains 9% chelated and absorbable iron for the plant in pH 3 to 11, is fully soluble in water and designed according to advanced chelate technology. NPK Nano—Fertilizer 20-20-20: powder, fully soluble in water and usable as spraying solution with a ratio of two per one thousand, and is environmentally friendly.

The seeds were collected from a local landrace in the south of Western Azerbaijan Province. The blocks were developed on the farm on 27 November 2017. After autumn plowing and land leveling, the sowing rows were created. The experimental plots were set at 6 m^2^. The winter sowing was performed on 28 November 2017 by the row sowing technique. On-row spacing was set at 1 cm and between-row spacing at 25 cm. The spring sowing was conducted on 27 February 2018. The thinning, gap-filling, and weeding operations were performed conventionally during the growing season. To determine the morphological traits at the harvest time, ten plants were randomly harvested from each plot to assess these traits. To estimate the yield, two side rows and 0.5 m from both ends of the plots were eliminated as the marginal effect. On 3 June 2018 plants reached their physiological maturity. Seed yield per plant was determined on ten plants randomly selected from each plot at physiological maturity.

According to the European Pharmacopoeia, the essential oil of *L. iberica* was extracted by distillation with water using a Clevenger [64]. Then, essential oil percentage was determined by the weight method and it was placed in the following formula to give essential oil yield:

Essential oil yield = Essential oil percentage × Seed yield(1)

Mucilage was measured using Kalyanasundaram et al.’s method [65]. Mucilage yield per unit area, which is a function of mucilage percentage and seed yield, was obtained from the following equation:

Mucilage yield = Mucilage percentage × Seed yield(2)

To perform antioxidant assays, a methanolic extract was first prepared from the samples. Then, the total flavonoid and phenol content of the extracts was determined using the method found in Marinova et al. [66] and Sakanaka et al. [67]. Ling and Zhao’s method was used to find out superoxide radical scavenging percentage [68]. To estimate nitric oxide radical scavenging percentage, the Garrat method was employed [69]. 1,1-diphenyl-2-picrylhydrazyl (DPPH) radical scavenging percentage was also measured by the procedure described in Brand-Williams et al. [70]. This method, with some modifications, was also used to measure chain-breaking activity using the DPPH reagent [70].

Data were statistically processed by analysis of variance (ANOVA) using the SAS 9.2 software package, and the means were compared by Duncan’s multiple range test (DMRT) at the *p* < 0.05 level.

## 5. Conclusions

The results showed that winter sowing outperformed spring sowing in terms of most quantitative and qualitative traits of *L. iberica*, owing to the better establishment in soil and more optimal use of environmental conditions. Also, the results for the nutrition of the plants by fertilizer regimes revealed that incorporated NPK-s + NPK-n + Fe-chelated-n was the most effective in influencing the quantitative and qualitative traits, but it did not show significant differences to NPK-n + Fe-chelated-n in most recorded traits. So, NPK-n + Fe-chelated-n can be presented as the best fertilization treatment for winter sowing, which will contribute to achieving the goals of sustainable agriculture, as incorporated NPK-s is eliminated.

## Figures and Tables

**Table 1:** Soil characteristics at the study site.

**EC ds m^−1^**	**pH**	**Texture**	**Clay**	**Silt**	**Sand**	**CaCO_3_**		**SP ^1^**
1.38	7.79	Clay loam	41%	36%	23%	15.71%		54
**N**		**Organic Carbon**	**Mn**	**B**	**Zn**	**Fe**	**K**	**P**
			mg kg^−1^					
0.03%		1.16%	11.2	0.28	1.1	8.11	282	9.02

^1^ Base saturation degree.

**Table 2:** Climatic conditions of the study site.

Month	Year	Monthly Precipitation (mm)	Mean Monthly Temperature (°C)	Mean Relative Humidity (%)
Dec-Jan	2017–2018	4.4	−4.4	63.2
Jan-Feb	2018	39.3	−4.2	63.1
Feb-Mar	2018	20.4	6.3	60.1
Mar-Apr	2018	59.9	11.6	54.7
Apr-May	2018	11.9	17.6	57.3
May-Jun	2018	0	22.7	51.4
Jun-Jul	2018	0.1	26.3	42.1
Jul-Aug	2018	0.6	25.2	50.1
Aug-Sept	2018	0	21.1	62
Sept-Oct	2018	1.8	12.6	73.1
Oct-Nov	2018	38.4	6.3	70.6
Nov-Dec	2018	6.8	1.7	50.7
